# Females and Males Show Differences in Early-Stage Transcriptomic Biomarkers of Lung Adenocarcinoma and Lung Squamous Cell Carcinoma

**DOI:** 10.3390/diagnostics11020347

**Published:** 2021-02-19

**Authors:** Quewang Liu, Yueying Wang, Meiyu Duan, Yusi Fan, Xingyuan Pan, Shuai Liu, Qiong Yu, Lan Huang, Fengfeng Zhou

**Affiliations:** 1 College of Computer Science and Technology, Jilin University, Changchun 130012, China; liuqw18@mails.jlu.edu.cn (Q.L.); Wyy18@mails.jlu.edu.cn (Y.W.); dmy235813@163.com (M.D.); panxingyuan209@gmail.com (X.P.); lshuai19@mails.jlu.edu.cn (S.L.); huanglan@jlu.edu.cn (L.H.); 2 Key Laboratory of Symbolic Computation and Knowledge Engineering of Ministry of Education, Jilin University, Changchun 130012, China; fan_yusi@163.com; 3 Department of Epidemiology and Biostatistics, School of Public Health, Jilin University, Changchun 130012, China; yuqiong@jlu.edu.cn; 4 College of Software, Jilin University, Changchun 130012, China

**Keywords:** sex disparity, early stage, transcriptomic biomarker, LUAD, LUSC

## Abstract

The incidence and mortality rates of lung cancers are different between females and males. Therefore, sex information should be an important part of how to train and optimize a diagnostic model. However, most of the existing studies do not fully utilize this information. This study carried out a comparative investigation between sex-specific models and sex-independent models. Three feature selection algorithms and five classifiers were utilized to evaluate the contribution of the sex information to the detection of early-stage lung cancers. Both lung adenocarcinoma (LUAD) and lung squamous cell carcinoma (LUSC) showed that the sex-specific models outperformed the sex-independent detection of early-stage lung cancers. The Venn plots suggested that females and males shared only a few transcriptomic biomarkers of early-stage lung cancers. Our experimental data suggested that sex information should be included in optimizing disease diagnosis models.

## 1. Introduction

Lung cancer is one of the most common malignant cancer types in both males and females [1,2,3]. It causes very high rates of morbidity and mortality in both sexes and is ranked as the most common cause of cancer-related deaths in the United States and other countries [4]. Non-small cell lung cancer (NSCLC) is the highest-occurring lung cancer and consists of two main subtypes, i.e., lung adenocarcinoma (LUAD) and squamous cell carcinoma (LUSC) [5].

Various diagnosis technologies have been developed to detect and determine the developmental stages of lung cancers [6,7]. The survival time of a lung cancer patient is associated with population genetic heterogeneity, inconsistent staging, anatomic variability and dissimilar tumor morphology [8]. The developmental stage at diagnosis is an essential factor to estimate the survival time of a lung cancer patient [9]. For example, NSCLC accounts for about 80% of all primary lung cancers and 60% of them are at the advanced stages III-B or IV at diagnosis [10].

Some studies show that males are more likely to be diagnosed at the stages III–IV than female patients [11]. The early diagnosis of NSCLC is essential to reduce the high mortality rate of lung cancer patients [12,13]. The early-stage NSCLC patients are mostly diagnosed by clinical imaging technologies like chest X-ray and CT, and results also show that males tend to have a higher rate of lung cancers than females [14]. Studies have suggested that the lung cancer mortality rate could be reduced via low-dose chest CT screening of the early-stage patients accompanied with appropriate treatments [15,16].

Various molecular biomarkers have been developed over the past few years and have demonstrated very promising performances in the diagnosis of lung cancers [17]. Molecular biomarkers together with artificial intelligent (AI) models provide accurate risk assessment, diagnosis, prognosis and personalized treatment decisions for lung cancer patients at any developmental stage [18]. As the lung tumor grows in the human body, cancer cells release large amounts of DNA, proteins and metabolites, which may serve as the discriminating biomarkers of lung cancers [19]. Female lung cancer patients tend to have genetic abnormalities in EGFR and ALK, etc., while the male patients tend to have mutated versions of KRAS and BRAF, etc. [20]. Although the diagnosis technologies have been substantially innovated and improved, many lung cancer patients are diagnosed when the disease is already in the advanced stages [21,22,23].

This study introduced sex information into the detection model of early-stage lung cancers and hypothesized that the sex-specific models may deliver better detection performances of early-stage lung cancers. This hypothesis was supported by comprehensive evaluations on the transcriptomic datasets of both LUAD and LUSC samples. The optimized biomarkers also demonstrated strong connections with the sex-specific biological functions.

## 2. Materials and Methods

### 2.1. Datasets

NCI’s Center for Cancer Genomics provided the cancer research community with a rich set of cancer genomics and clinical data through an efficient and standardized workflow called the genome characterization pipeline [24]. The transcriptomic datasets and the sex information of the two cancer types LUAD and LUSC were retrieved from the public repository, The Cancer Genome Atlas (TCGA) [25,26].

A sample was kept for the investigations in this study if the sample had the transcriptomic data, developmental stage and sex information. The LUAD and LUSC stage system in the TCGA dataset was based on the size of the primary tumor (T), the spread of cancer to lymph nodes (N) and distant metastasis (M) according to the American Joint Committee on Cancer [27]. We merge stage I, stage IA and stage IB into stage I. We merge stage II, stage IIA and stage IIB into stage II. We also merge stage III A and stage III B into stage III. As shown in Table 1, the TNM (Tumor size, lymph Node metastasis, distant Metastasis) stage is used to define the stage. In the absence of specific information about TNM staging, we use pathological staging directly in the clinical literature. 

There were 273, 120, 84 and 26 LUAD samples in the four stages I, II, III and IV, respectively. The first two stages I and II were grouped as the early stage and the other two stages III and IV were regarded as the advanced stage [28]. There were 244, 162, 84 and 7 LUSC samples in the four stages I, II, III and IV, respectively. So, there were 406 early-stage and 91 advanced-stage LUSC samples. In total, this study investigated 1000 lung cancer samples in the two major subtypes LUAD and LUSC.

The gastric cancer dataset was also retrieved from the TCGA database as an independent validation of our hypothesis [29]. The same sample screening procedure was carried out. There were 59, 130, 183 and 44 gastric cancer samples of the stages I, II, III and IV, respectively. Therefore, a binary classification dataset of the 189 early-stage and 227 advanced-stage gastric cancer samples was established.

### 2.2. Feature Selection and Classification Algorithms

Three feature selection algorithms were used to select the biomarkers in a specific dataset. Support vector machine (SVM)-based recursive feature elimination (SVM-RFE) evaluated the features’ coefficients in the SVM model and recursively eliminated the features with small coefficients [28,30]. The least absolute shrinkage and selection operator (LASSO) was a regression-based feature selection algorithm and selected the features by assigning non-zero weights to these chosen features, and the features may be ranked in the descendent order of their weights [31,32]. *T*-test (Ttest) was widely used to test the statistical associations of the features with the class label, and selected the top-ranked features with the best classification performances [33].

Different classifiers may perform differently on a dataset and a disease diagnosis study usually delivers the best model. So, this study evaluated a given feature subset using five representative classifiers and the best accuracy achieved in these five classifiers was used to measure this feature subset. The five classifiers were Logistic Regression (LR), Support Vector Machine (SVM), Random Forest (RF), AdaBoost (Ad) and Gaussian Naïve Bayesian (GNB) [34].

The LR model took the natural logarithm of the odds as a regression function of the predictors and it is a popular technique used in machine learning to construct classification models [35,36]. The purpose of SVM is to create a decision boundary between two categories that can predict the label based on one or more feature vectors [37,38]. RF can take care of different types of data imbalance and has the ability to efficiently handle nonlinear classification tasks [39]. Ad is a popular ensemble method that combines several weak learners to boost generalization performance [40]. GNB assumes that all functions are analyzed independently of each other [41]. A 10-fold cross validation strategy (10FCV) was used to calculate the classification performances. 10FCV referred to the 10-fold cross-validation strategy. In summary, the dataset was randomly split into 10 equally sized sub-datasets. On each cross-validation iteration, nine sub-datasets were used to train a model and the remaining one sub-dataset was employed as the test set [42]. The overall prediction result was calculated through the results of 10 iterations.

### 2.3. Performance Evaluation Metrics

This study conducted a series of evaluation experiments to demonstrate that sex information is essential to detect early-stage lung cancers. The investigated problem setting was the binary classification problem. This study evaluated a binary classification model using the detection accuracy (Acc), sensitivity (Sn) and specificity (Sp), the same as in References [43,44]. A binary classification tried to build a classification model to discriminate the positive and negative samples. Sn and Sp were defined as the percentages of the correctly predicted positive and negative samples, respectively. The detection accuracy (Acc) was defined as the percentage of correct samples.

### 2.4. Programming and Running Environments

The experiments in study were carried out on a Windows 10 computer with 8 GB system memory and one Intel Core i5-8250U CPU. All the experiments were programmed using Python version 3.6.5 and scikit-learn version 0.19.1.

### 2.5. Workflow of This Study

**Figure 1:**
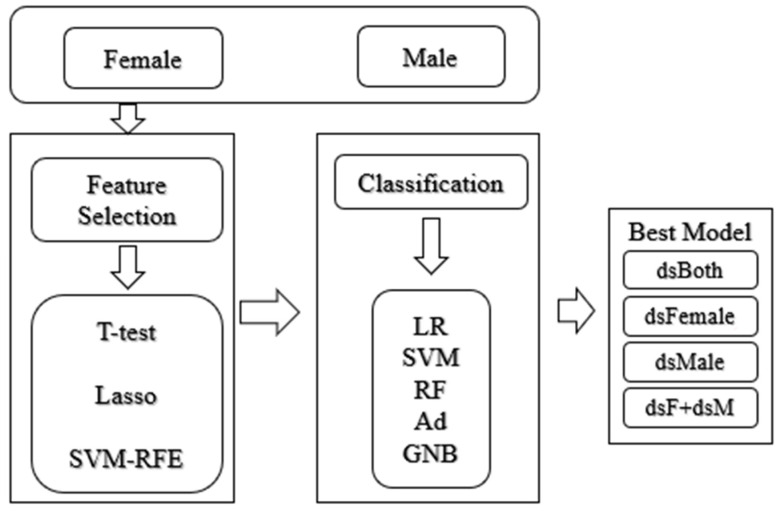
Experimental workflow of this study. Three feature selection algorithms were evaluated for their binary classification performances using five classifiers on the datasets with both sexes. The three feature selection methods were Ttest, least absolute shrinkage and selection operator (LASSO) and support vector machine (SVM)-based recursive feature elimination (SVM-RFE). The five classifiers were Logistic Regression (LR), Support Vector Machine (SVM), Random Forest (RF), AdaBoost (Ad) and Gaussian Naïve Bayesian (GNB).

The experiments were carried out in the following workflow, as shown in Figure 1. Each cancer subtype consisted of four datasets. Firstly, the datasets of male and female samples were denoted as dsMale and dsFemale, respectively. The 10FCV classification performance of the classifier “C” was calculated on dsMale and denoted as C(dsMale). The notation C(dsFemale) was defined in the same way. The combined dataset dsBoth=dsFemale∪dsMale. The performance of the classifier “C” was calculated using the 10FCV on the dataset dsBoth and the performance was denoted as C(dsBoth). This study sought to investigate whether the duet of the separately built sex-specific classification models may outperform the model without considering the sex information. Therefore, the notation “dsF+dsM” referred to the classification performance of all the samples using the sex-specific models C(dsFemale) or C(dsMale).

## 3. Results

### 3.1. Baseline Summary of the Two Lung Cancer Subtypes

The samples of both sexes in the four developmental stages are summarized in Table 2. We firstly evaluated the null hypothesis that the sex information of the samples is associated with the tumor stage. Chi-squared test was used to measure the statistical significance of the association between sex and stage. There were no significant differences in males and females in the tumor stages in LUAD (*p* = 0.075) and LUSC (*p* = 0.682). The Spearman correlation coefficient (SCC) was used to measure the correlation between sex and stage of the samples. The correlation between sex and stage in LUAD was −0.082 (*p* = 0.067), while the SCC was −0.050 (*p* = 0.267) in LUSC. Therefore, we did not find correlations between sex and tumor stages in either the LUAD or LUSC samples.

### 3.2. Evaluation of the Classifiers on the Ttest-Ranked Features

**Figure 2:**
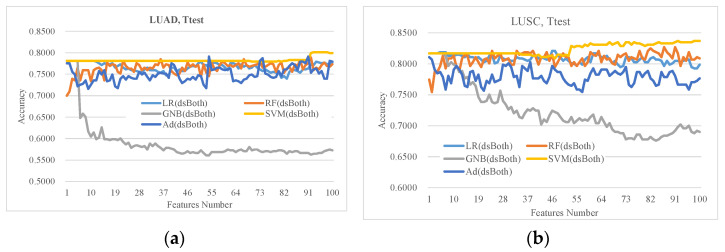
Classification performances of the top-ranked 100 features by Ttest. The classification accuracy was calculated by the 10-fold cross validation strategy for the five classifiers, i.e., LR, SVM, RF, GNB, Ad. The horizontal axis is the number of the top-ranked features used in each calculation and the vertical axis is the classification accuracy. The experimental data were visualized (**a**) on the LUAD, and (**b**) on the LUSC.

Ttest was widely used to evaluate the associations of various biomedical features with the phenotypes. This study used the Ttest-ranked top 100 features to evaluate how the five representative classifiers performed on a given feature subset. Figure 2 illustrated that the classifier GNB did not perform well on the Ttest-ranked features on both LUAD and LUSC datasets. The classifiers LR and SVM performed similarly well when using a few features. As more features were used, SVM outperformed the other classifiers in most cases. SVM also achieved the best accuracy Acc = 0.8012 using 93 features on the LUAD dataset dsBoth, which was higher than those of all the other four classifiers. The same pattern was observed on the LUSC dataset dsBoth. SVM achieved the best accuracy Acc = 0.8370 using 92 features, and outperformed the other four classifiers using any number of features. Therefore, the following sections use SVM as the evaluation classifier.

### 3.3. Sex Disparities Using the Ttest-Ranked Biomarkers

**Figure 3:**
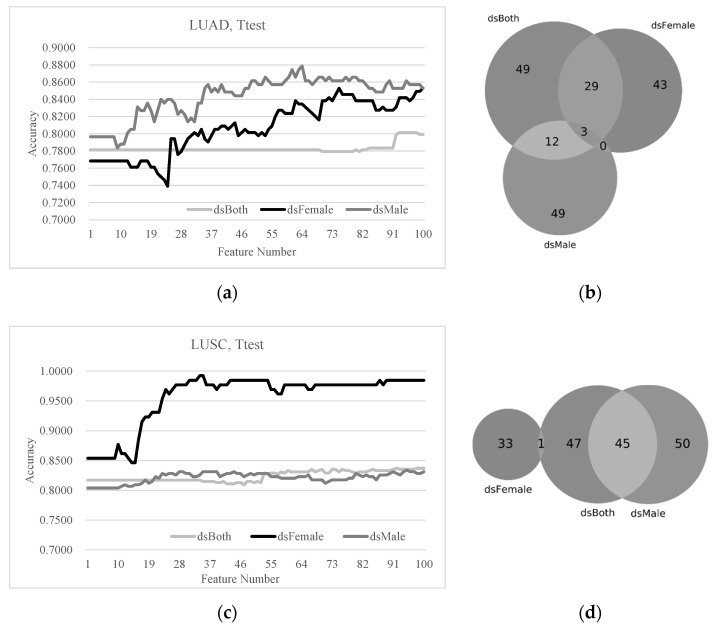
The SVM (support vector machine) prediction performances and the sex disparities of the top-100 Ttest-ranked biomarkers. (**a**) The prediction performances of the classifier SVM and (**b**) the Venn plot of the Ttest-ranked biomarkers on the three models dsBoth, dsFemale and dsMale of the lung cancer subtype LUAD. (**c**) The prediction performances of the classifier SVM and (**d**) the Venn plot of the Ttest-ranked biomarkers on the three models dsBoth, dsFemale and dsMale of the lung cancer subtype LUSC.

A comparison of the early-stage detection models and the Ttest-ranked biomarkers was carried out on the two lung cancer subtypes, as shown in Figure 3. Figure 3a shows that the best model on the LUAD dsBoth dataset achieved the accuracy Acc = 0.8012 using 93 features, while only 30 features were needed to achieve the same Acc on the LUAD dsFemale dataset. Actually, both the dsFemale (Acc = 0.8529 and 75 features) and dsMale (Acc = 0.8788 and 64 features) datasets can be classified with better detection accuracies and fewer features. The Venn plot in Figure 3b shows that the female LUAD patients had 43 unique biomarkers, which were not observed in either dsMale and dsBoth, while the male LUAD patients had 49 such unique biomarkers, which were not biomarkers in the dsFemale and the dsBoth datasets. There were only three early-stage LUAD biomarkers shared by both dsFemale and dsMale.

The dsMale dataset had similar performances in early-stage lung cancer detection as the dsBoth dataset, as shown in Figure 3c, while the SVM model using the Ttest-ranked biomarkers outperformed the models of both dsMale and dsBoth using any number of features. The data supported the existence of the sex disparities in the early-stage detection performances of the LUSC samples. The Venn plot illustrated that the dsFemale and dsMale datasets did not share any Ttest-ranked biomarkers.

### 3.4. Sex Disparities in the Biomarkers Ranked by LASSO and SVM-RFE

**Figure 4:**
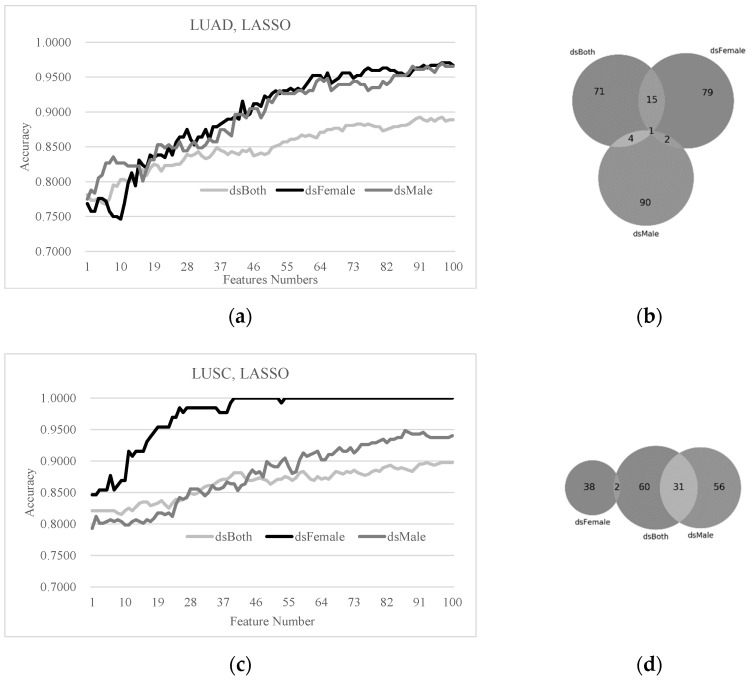
The SVM prediction performances and the sex disparities of the top-100 LASSO-ranked biomarkers. (**a**) The prediction performances of the classifier SVM and (**b**) the Venn plot of the LASSO-ranked biomarkers on the three models dsBoth, dsFemale and dsMale of the lung cancer subtype LUAD. (**c**) The prediction performances of the classifier SVM and (**d**) the Venn plot of the LASSO-ranked biomarkers on the three models dsBoth, dsFemale and dsMale of the lung cancer subtype LUSC.

The LASSO-ranked biomarkers were also compared for sex disparity in the detection models of early-stage lung cancers, as shown in Figure 4. The LASSO-ranked biomarkers achieved very good detection accuracies of early-stage LUAD and LUSC samples. The best detection model of the LUAD dsBoth dataset reached Acc = 0.8926 using 91 features, as shown in Figure 4a. Both of the best models of the LUAD dsFemale and dsMale outperformed the above model with accuracy improvements 0.0779 and 0.0771, respectively. The best detection accuracy Acc = 0.8974 of the LUSC dsBoth dataset (Figure 4c) was also improved by 0.1026 and 0.0508 using the LUSC dsFemale and dsMale datasets, respectively. The best model of the LUSC dsFemale dataset even reached Acc = 1,0000 using only 40 features. The overlap between the LUAD dsFemale and dsMale biomarkers detected by LASSO was only three genes (Figure 4b). No overlap was observed between the LUSC dsFemale and dsMale biomarkers (Figure 4d).

**Figure 5:**
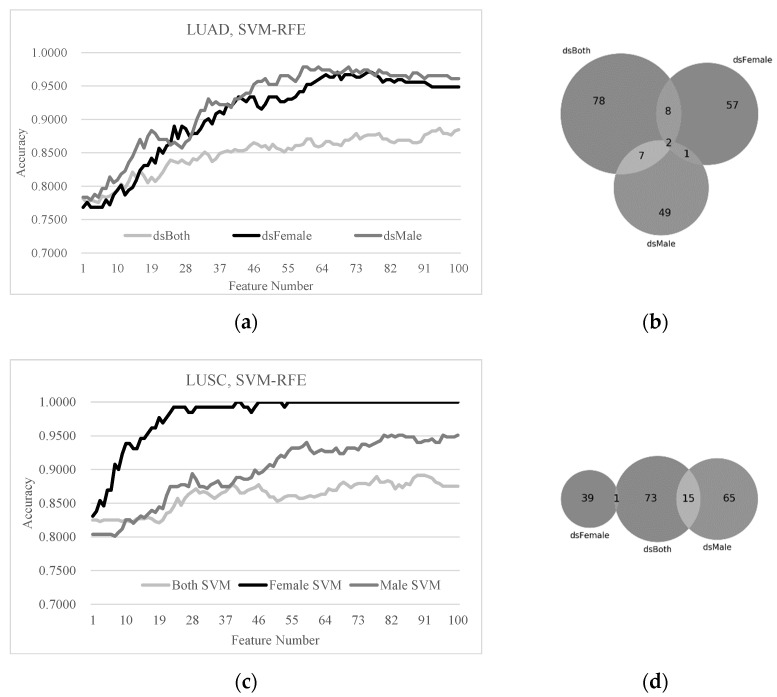
The SVM prediction performances and the sex disparities of the top-100 SVM-RFE-ranked biomarkers. (**a**) The prediction performances of the classifier SVM and (**b**) the Venn plot of the SVM-RFE-ranked biomarkers on the three models dsBoth, dsFemale and dsMale of the lung cancer subtype LUAD. (**c**) The prediction performances of the classifier SVM and (**d**) the Venn plot of the SVM-RFE-ranked biomarkers on the three models dsBoth, dsFemale and dsMale of the lung cancer subtype LUSC.

The similar patterns were further confirmed by an additional feature selection algorithm SVM-RFE, as shown in Figure 5. The detection accuracies of the early-stage LUAD and LUSC patients were much higher using the sex-specific models than the mixture of both sexes. None or a negligible number of genes were observed to be shared by the female- and male-specific biomarkers of the early-stage LUAD and LUSC patients.

### 3.5. Sex-Specific Models May Improve Early-Stage Lung Cancer Detection

**Figure 6:**
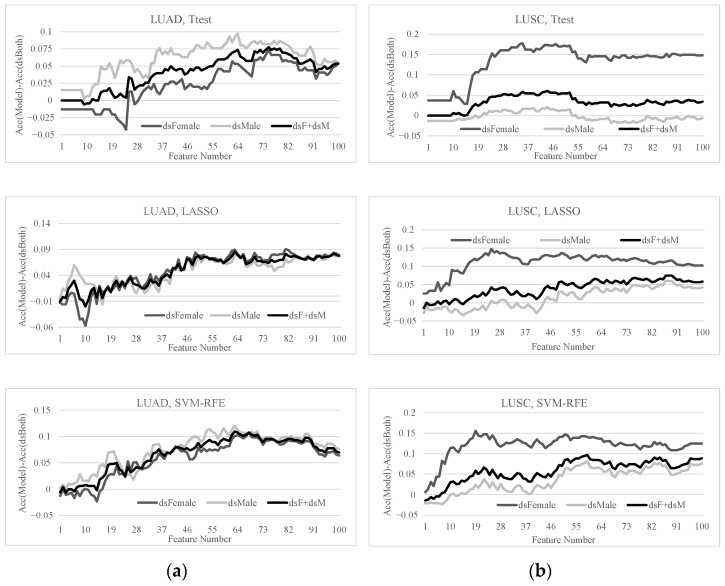
SVM classification performances of the top 100 features in LUAD and LUSC. The features were ranked by Ttest, LASSO and SVM-RFE, respectively. The horizontal axis gives the number of features used in each data point. The vertical axis gives the detection accuracy of each model minus that of the dsBoth dataset. The improvements of the datasets dsFemale/dsMale/dsF+dsM were illustrated for the two lung cancer subtypes (**a**) LUAD and (**b**) LUSC.

This section quantitatively investigated how the detection of early-stage lung cancers may be improved by the sex-specific models, as shown in Figure 6. The previous sections illustrated that the sex-specific detection models of early-stage lung cancers outperformed the detection model using samples from both sexes. Therefore, we formulated the problem setting as the early-stage detection of a given lung cancer sample using the sex-specific model, and calculated the overall detection accuracy of the early-stage lung cancers. This problem setting was denoted as “dsF+dsM”. A positive value in Figure 6 indicated that the specific model outperformed the detection model using the dsBoth dataset, i.e., building a detection model using both female and male samples.

Figure 6 illustrated that the sex-specific models outperformed the models using the dsBoth dataset in most cases. The detection model using the LUAD dsBoth dataset may be improved by 0.1093 and 0.0855 in Acc using 63 features selected by SVM-RFE and LASSO, respectively. The feature selection algorithm SVM-RFE improved the model using the LUSC dsBoth dataset by 0.0966 in Acc using 59 features.

Overall, the sex-specific models significantly improved on the conventional detection model of early-stage lung cancers for both sexes.

### 3.6. Independent Evaluation of the Hypothesis on Gastric Cancer

**Figure 7:**
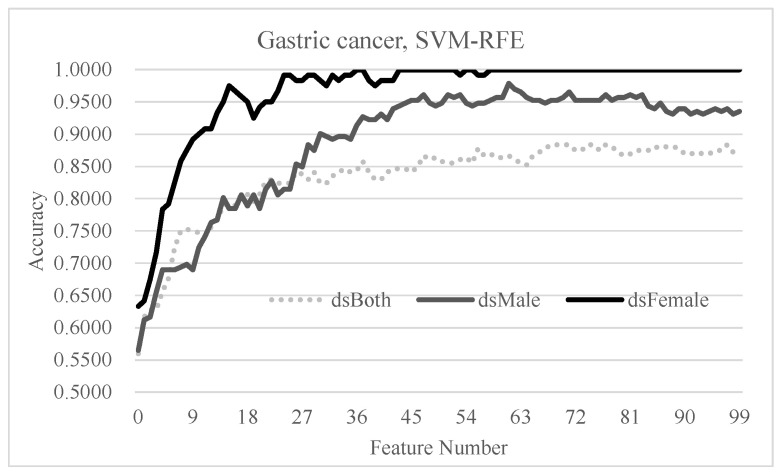
Performance evaluation of the sex-specific detection models of early-stage gastric cancers. The horizontal axis gives the number of features used in each data point. The vertical axis gives the detection accuracy of each model. The models dsFemale and dsMale were for the female- and male-specific models. The models of the dsBoth dataset were optimized using samples from both sexes.

The gastric cancer patients were further evaluated as independent proof for our hypothesis that the sex-specific models may improve on the conventional model using samples of both sexes, as shown in Figure 7. The highest detection accuracy of the dsBoth dataset was Acc = 0.8835 using 68 features. However, the sex-specific models achieved the best accuracies of 1.0000 using 36 features and 0.9784 using 61 features for the female and male samples, respectively. 

## 4. Discussion

The purpose of this study was to evaluate whether separating gender in LUSC and LUAD can improve the detection of early-diagnosis biomarkers. Several studies have shown that there are sex differences in lung cancer regarding incidence and mutation status [45,46]. However, few studies have considered gender differences when analyzing early tumor diagnosis. In this study, we analyzed the gender differences in gene expression in early and advanced LUSC and LUAD. In addition, we used three feature selection methods combined with SVM machine learning to analyze whether there were gender-specific early diagnosis biomarker sets. Our findings showed that gender-specific models significantly improved the sex-independent detection models of early lung cancer.

This study showed that in the female sample, the accuracy of early prediction was higher than that of the male samples and the total samples. The possible reasons were as follows. Firstly, in relation to diagnosis interval and stage of cancer diagnosis, it was found that women had longer diagnostic intervals and women tended to get diagnosed at an earlier stage [47]. Secondly, cigarette smoking is the major pathogenic factor for lung cancer [48,49]. Although the greatest risk of lung cancer is smoking, factors like age, radon exposure, environmental pollution, occupational exposures, gender, race and pre-existing lung disease are also important contributors [50]. Azagba’s study showed that from 2011 to 2018, over time, heavy smoking decreased significantly among female students but increased significantly among male students, and male youth smoked more heavily and started smoking earlier [51]. Lastly, the incidence of lung cancer among non-smokers is on the rise, which can be attributed to environmental and occupational exposure to various kinds of hazardous substances, and some occupations have a higher risk of lung cancer, such as bartenders, ceramics industry, coal gasification and coke production, truck drivers, construction industry, rubber industry and uranium mining [52]. Occupational exposure to organic dust was associated with increased lung cancer risk in a large pooled case-control study [53]. The study by Suraya et al. found that in each section of the Indonesian Standard of Industrial Classification 2015, compared with workers in other sectors, construction workers had a higher risk of lung cancer, and the proportion of males was dominant in work areas such as mining, quarrying and construction [54]. Eguchi et al. found that for Japanese men, mining, electricity and gas, fisheries and agriculture and forestry had the higher mortality risks for lung, gastric and colorectal cancers [55].

The study had the following limitations. First of all, this study was a retrospective study and the uninvestigated variables in the dataset could not be controlled. Secondly, our current research was carried out on the TCGA database, which provided patients’ transcriptome data sets. It can be expected that a future study with more controlled clinical data (such as smoking and occupation) will help make the hypothesis of this study more convincing.

## 5. Conclusions

This study comprehensively evaluated sex-specific detection models of early-stage lung cancer and gastric cancer. The experimental data strongly suggested that the detection of early-stage lung cancer may be substantially improved by simply using the sex-specific models. 

Sex is different from the data types of many OMIC values and is difficult to be directly integrated with the OMIC data to build a prediction model. This study demonstrated that a simple system of using the sex-specific models to detect early-stage cancers may improve the conventional sex-independent models. Efficient integrated modeling technologies will be investigated in future work.

## Figures and Tables

**Table 1:** Stage according to The TNM (Tumor size, lymph Node metastasis, distant Metastasis) Staging System.

	N0	N1	N2	N3	M1a	M1b
T1	I	II	III	III	IV	IV
T1a	I	II	III	III	IV	IV
T1b	I	II	III	III	IV	IV
T2	I	II	III	III	IV	IV
T2a	I	II	III	III	IV	IV
T2b	II	II	III	III	IV	IV
T3	II	III	III	III	IV	IV
T4	III	III	III	III	IV	IV

T1, T2, T3, T4: Refers to the size and/or extent of the main tumor. The higher the number after the T, the larger the tumor or the more it has grown into nearby tissues. T’s may be further divided to provide more detailed sub-stages, such as T1a and T1b. N1, N2, N3: Refers to the number and location of lymph nodes that contain cancer. The higher the number after the N, the more lymph nodes that contain cancer. M1: Cancer has spread to other parts of the body.

**Table 2:** Baseline data of the LUAD (lung adenocarcinoma) and LUSC (squamous cell carcinoma) samples in the TCGA (The Cancer Genome Atlas) database. The numbers in this table are the numbers of samples in each group of samples.

		Stage I	Stage II	Stage III	Stage IV
LUAD	Male	113	66	38	14
	Female	160	54	46	12
LUSC	Male	175	122	64	6
	Female	69	40	20	1

## Data Availability

The data presented in this study are publicly available at the TCGA database https://portal.gdc.cancer.gov/.

## References

[1] Prabhakar B., Shende P., Augustine S. (2018). Current trends and emerging diagnostic techniques for lung cancer. Biomed. Pharmacother..

[2] Tomasetti M., Amati M., Neuzil J., Santarelli L. (2017). Circulating epigenetic biomarkers in lung malignancies: From early diagnosis to therapy. Lung Cancer.

[3] Shi Y.B., Li J., Lai X.N., Jiang R., Zhao R.C., Xiong L.X. (2020). Multifaceted Roles of Caveolin-1 in Lung Cancer: A New Investigation Focused on Tumor Occurrence, Development and Therapy. Cancers.

[4] Lababede O., Meziane M., Rice T. (2011). Seventh edition of the cancer staging manual and stage grouping of lung cancer: Quick reference chart and diagrams. Chest.

[5] Yang Y., Wang M., Liu B. (2019). Exploring and comparing of the gene expression and methylation differences between lung adenocarcinoma and squamous cell carcinoma. J. Cell. Physiol..

[6] Nasim F., Sabath B.F., Eapen G.A. (2019). Lung Cancer. Med Clin. N. Am..

[7] Serke M., Schönfeld N. (2007). Diagnosis and staging of lung cancer. Dtsch. Med. Wochenschr..

[8] Nesbitt J.C., Putnam J.B., Walsh G.L., Roth J.A., Mountain C.F. (1995). Survival in early-stage non-small cell lung cancer. Ann. Thorac. Surg..

[9] Smith C.B., Bonomi M., Packer S., Wisnivesky J.P. (2011). Disparities in lung cancer stage, treatment and survival among American Indians and Alaskan Natives. Lung Cancer.

[10] Lam W.K., Tsang K.W., Ip M.S. (1998). Chemotherapy for advanced (stage IIIB and stage IV) non-small cell lung cancer: The Hong Kong perspective. Respirology.

[11] Tolwin Y., Gillis R., Peled N. (2020). Gender and lung cancer-SEER-based analysis. Ann. Epidemiol..

[12] Shende P., Augustine S., Prabhakar B., Gaud R.S. (2019). Advanced multimodal diagnostic approaches for detection of lung cancer. Expert Rev. Mol. Diagn..

[13] Cykert S., Eng E., Walker P., Manning M.A., Robertson L.B., Arya R., Jones N.S., Heron D.E. (2019). A system-based intervention to reduce Black-White disparities in the treatment of early stage lung cancer: A pragmatic trial at five cancer centers. Cancer Med..

[14] Doria-Rose V.P., White M.C., Klabunde C.N., Nadel M.R., Richards T.B., McNeel T.S., Rodriguez J.L., Marcus P.M. (2012). Use of lung cancer screening tests in the United States: Results from the 2010 National Health Interview Survey. Cancer Epidemiol. Biomark. Prev..

[15] Taylor K.L., Deros D.E., Fallon S., Stephens J., Kim E., Lobo T., Davis K.M., Luta G., Jayasekera J., Meza R. (2019). Study protocol for a telephone-based smoking cessation randomized controlled trial in the lung cancer screening setting: The lung screening, tobacco, and health trial. Contemp. Clin. Trials.

[16] Aberle D.R., Adams A.M., Berg C.D., Black W.C., Clapp J.D., Fagerstrom R.M., Gareen I.F., Gatsonis C., Marcus P.M., Sicks J.D. (2011). Reduced lung-cancer mortality with low-dose computed tomographic screening. N. Engl. J. Med..

[17] Tufman A., Huber R.M. (2010). Biological markers in lung cancer: A clinician’s perspective. Cancer Biomark. Sect. A Dis. Markers.

[18] Sears C.R., Mazzone P.J. (2020). Biomarkers in Lung Cancer. Clin. Chest Med..

[19] Khanmohammadi A., Aghaie A., Vahedi E., Qazvini A., Ghanei M., Afkhami A., Hajian A., Bagheri H. (2020). Electrochemical biosensors for the detection of lung cancer biomarkers: A review. Talanta.

[20] Xue X., Asuquo I., Hong L., Gao J., Dong Z., Pang L., Jiang T., Meng M., Fan J., Wen J. (2020). Catalog of Lung Cancer Gene Mutations Among Chinese Patients. Front. Oncol..

[21] Bryan S., Masoud H., Weir H.K., Woods R., Lockwood G., Smith L., Brierley J., Gospodarowicz M., Badets N. (2018). Cancer in Canada: Stage at diagnosis. Health Rep..

[22] Campanella A., De Summa S., Tommasi S. (2019). Exhaled breath condensate biomarkers for lung cancer. J. Breath Res..

[23] Yousefi M., Ghaffari P., Nosrati R., Dehghani S., Salmaninejad A., Abarghan Y.J., Ghaffari S.H. (2020). Prognostic and therapeutic significance of circulating tumor cells in patients with lung cancer. Cell. Oncol..

[24] NCI’s Genome Characterization Pipeline. https://www.cancer.gov/about-nci/organization/ccg/research/genomic-pipeline#collection-processing..

[25] Zhao J., Bao W., Cai W. (2020). Immune Infiltration Landscape in Lung Squamous Cell Carcinoma Implications. Biomed. Res. Int..

[26] Li X., Shi Y., Yin Z., Xue X., Zhou B. (2014). An eight-miRNA signature as a potential biomarker for predicting survival in lung adenocarcinoma. J. Transl. Med..

[27] Sathipati S.Y., Ho S.Y. (2020). Novel miRNA signature for predicting the stage of hepatocellular carcinoma. Sci. Rep..

[28] Li C., Luo X., Qi Y., Gao Z., Lin X. (2020). A new feature selection algorithm based on relevance, redundancy and complementarity. Comput. Biol. Med..

[29] Feng X., Li J., Li H., Chen H., Li F., Liu Q., You Z.H., Zhou F. (2019). Age Is Important for the Early-Stage Detection of Breast Cancer on Both Transcriptomic and Methylomic Biomarkers. Front. Genet..

[30] Tian X.P., Su N., Wang L., Huang W.J., Liu Y.H., Zhang X., Huang H.Q., Lin T.Y., Ma S.Y., Rao H.L. (2020). A CpG Methylation Classifier to Predict Relapse in Adults with T-Cell Lymphoblastic Lymphoma. Clin. Cancer Res..

[31] Kang C., Huo Y., Xin L., Tian B., Yu B. (2019). Feature selection and tumor classification for microarray data using relaxed Lasso and generalized multi-class support vector machine. J. Theor. Biol..

[32] Huang Y.Q., Liang C.H., He L., Tian J., Liang C.S., Chen X., Ma Z.L., Liu Z.Y. (2016). Development and Validation of a Radiomics Nomogram for Preoperative Prediction of Lymph Node Metastasis in Colorectal Cancer. J. Clin. Oncol..

[33] Chen L., Li Q., Song H., Gao R., Yang J., Dong W., Dang W. (2020). Classification of schizophrenia using general linear model and support vector machine via fNIRS. Phys. Eng. Sci. Med..

[34] Giannini V., Rosati S., Defeudis A., Balestra G., Vassallo L., Cappello G., Mazzetti S., De Mattia C., Rizzetto F., Torresin A. (2020). Radiomics predicts response of individual HER2-amplified colorectal cancer liver metastases in patients treated with HER2-targeted therapy. Int. J. Cancer.

[35] LaValley M.P. (2008). Logistic regression. Circulation.

[36] Bonte C., Vercauteren F. (2018). Privacy-preserving logistic regression training. BMC Med. Genom..

[37] Huang S., Cai N., Pacheco P.P., Narrandes S., Wang Y., Xu W. (2018). Applications of Support Vector Machine (SVM) Learning in Cancer Genomics. Cancer Genom. Proteom..

[38] Sanz H., Valim C., Vegas E., Oller J.M., Reverter F. (2018). SVM-RFE: Selection and visualization of the most relevant features through non-linear kernels. BMC Bioinform..

[39] Paul A., Mukherjee D.P., Das P., Gangopadhyay A., Chintha A.R., Kundu S. (2018). Improved Random Forest for Classification. IEEE Trans. Image Process..

[40] Zhang P.B., Yang Z.X. (2018). A Novel AdaBoost Framework with Robust Threshold and Structural Optimization. IEEE Trans. Cybern..

[41] Chandak T., Mayginnes J.P., Mayes H., Wong C.F. (2020). Using machine learning to improve ensemble docking for drug discovery. Proteins.

[42] Tejera E., Carrera I., Jimenes-Vargas K., Armijos-Jaramillo V., Sánchez-Rodríguez A., Cruz-Monteagudo M., Perez-Castillo Y. (2019). Cell fishing: A similarity based approach and machine learning strategy for multiple cell lines-compound sensitivity prediction. PLoS ONE.

[43] Haenssle H.A., Winkler J.K., Fink C., Toberer F., Enk A., Stolz W., Deinlein T., Hofmann-Wellenhof R., Kittler H., Tschandl P. (2020). Skin lesions of face and scalp—Classification by a market-approved convolutional neural network in comparison with 64 dermatologists. Eur. J. Cancer.

[44] Csutak C., Ștefan P.A., Lupean R.A., Lenghel L.M., Mihu C.M., Lebovici A. (2020). Computed tomography in the diagnosis of intraperitoneal effusions: The role of texture analysis. Bosn. J. Basic Med. Sci..

[45] Bray F., Ferlay J., Soerjomataram I., Siegel R.L., Torre L.A., Jemal A. (2018). Global cancer statistics 2018: GLOBOCAN estimates of incidence and mortality worldwide for 36 cancers in 185 countries. CA Cancer J. Clin..

[46] Stapelfeld C., Dammann C., Maser E. (2020). Sex-specificity in lung cancer risk. Int. J. Cancer.

[47] Rana R.H., Alam F., Alam K., Gow J. (2020). Gender-specific differences in care-seeking behaviour among lung cancer patients: A systematic review. J. Cancer Res. Clin. Oncol..

[48] Hammerschmidt S., Wirtz H. (2009). Lung cancer: Current diagnosis and treatment. Dtsch. Arztebl. Int..

[49] Schwartz A.G., Cote M.L. (2016). Epidemiology of Lung Cancer. Adv. Exp. Med. Biol..

[50] de Groot P., Munden R.F. (2012). Lung cancer epidemiology, risk factors, and prevention. Radiol. Clin. N. Am..

[51] Azagba S., Manzione L., Shan L., King J. (2020). Trends in Smoking Behaviors Among US Adolescent Cigarette Smokers. Pediatrics.

[52] Shankar A., Dubey A., Saini D., Singh M., Prasad C.P., Roy S., Bharati S.J., Rinki M., Singh N., Seth T. (2019). Environmental and occupational determinants of lung cancer. Transl. Lung Cancer Res..

[53] Peters S., Kromhout H., Olsson A.C., Wichmann H.E., Brüske I., Consonni D., Landi M.T., Caporaso N., Siemiatycki J., Richiardi L. (2012). Occupational exposure to organic dust increases lung cancer risk in the general population. Thorax.

[54] Suraya A., Nowak D., Sulistomo A.W., Icksan A.G., Berger U., Syahruddin E., O’Reilly S.B. (2021). Excess Risk of Lung Cancer Among Agriculture and Construction Workers in Indonesia. Ann. Glob. Health.

[55] Eguchi H., Wada K., Prieto-Merino D., Smith D.R. (2017). Lung, gastric and colorectal cancer mortality by occupation and industry among working-aged men in Japan. Sci. Rep..

